# Metallothionein in human oesophagus, Barrett's epithelium and adenocarcinoma

**DOI:** 10.1038/sj.bjc.6600473

**Published:** 2002-08-27

**Authors:** P Coyle, G Mathew, P A Game, J C Myers, J C Philcox, A M Rofe, G G Jamieson

**Affiliations:** Division of Clinical Biochemistry, Institute of Medical and Veterinary Science, Frome Road, Adelaide, SA 5000 Australia; Surgery 1, Christian Medical College and Hospital, Vellore, 632004, South India; Department of Surgery, University of Adelaide, Adelaide, SA 5005 Australia

**Keywords:** metallothionein, Barrett's oesophagus, adenocarcinoma, risk factors

## Abstract

The potential of the metal-binding protein, metallothionein, in assessing the progression of normal oesophagus through Barrett's to adenocarcinoma was investigated. Metallothionein was quantitatively determined in resected tissues from patients undergoing oesophagectomy for high grade dysplasia/adenocarcinoma and in biopsies from patients with Barrett's syndrome. In 10 cancer patients, metallothionein concentrations in adenocarcinoma were not significantly different from normal oesophagus, although six had elevated metallothionein concentrations in the metaplastic tissue bordering the adenocarcinoma. In 17 out of 20 non-cancer patients with Barrett's epithelium, metallothionein was significantly increased by 108% (*P*<0.004). There was no association between the metallothionein levels in Barrett's epithelium and the presence of inflammatory cells, metaplasia or dysplasia. Metallothionein is a marker of progression from normal to Barrett's epithelium but is not increased in oesophageal adenocarcinoma.

*British Journal of Cancer* (2002) **87**, 533–536. doi:10.1038/sj.bjc.6600473 www.bjcancer.com

© 2002 Cancer Research UK

## 

Chronic irritation of the oesophagus by reflux of acidic gastric juices can lead to Barrett's oesophagus which is characterised by columnar metaplasia which replaces the normal stratified squamous epithelium in the lower third of the oesophagus. Barrett's oesophagus is considered to be a premalignant lesion and in some patients leads to adenocarcinoma of the oesophagus, a cancer with poor prognosis and of increasing prevalence in the Western World (Jankowski *et al*, 1999; Krishnadath *et al*, 2001). It is generally accepted that cancer evolves from Barrett's oesophagus by progression from metaplasia, to low-grade dysplasia, high-grade dysplasia and then to adenocarcinoma. Thus, biopsy with histological demonstration of high grade dysplasia provides the main evidence for a transition from Barrett's to adenocarcinoma (Ortiz-Hidalgo *et al*, 1998; Krishnadath *et al*, 2001). However the staging of Barrett's oesophagus is subject to considerable inter- and intraobserver variation. Consequently interest is mounting in a range of biological markers that may help to support histological diagnosis and improve the efficacy of surveillance programs (Krishnadath *et al*, 2001).

Metallothionein (MT) is a low molecular weight, cysteine-rich protein, with metal-binding and antioxidant properties. It is rapidly induced by a variety of agents including inflammatory cytokines, hormones and cytotoxic agents. Although the primary role of MT is controversial, it is known to regulate Zn homeostasis and be involved in cellular defence mechanisms. Its ability to donate Zn to many Zn-requiring enzymes and transcription factors suggest a role for MT in the processes of cell proliferation and differentiation (reviewed by Cherian *et al*, 1994; Coyle *et al*, 2002). MT is expressed in a variety of human cancers where it has been found to correlate with proliferative activity, tumour cell progression and resistance to anti-cancer drugs (Cherian *et al*, 1994). Increased MT expression has been found in squamous cell carcinomas of the oesophagus (Hishikawa *et al*, 1999), serous ovarian tumours (Tan *et al*, 1999), breast carcinomas (Fresno *et al*, 1993), astrocytomas (Hiura *et al*, 1998) and bladder cancers (Sens *et al*, 2000). High MT expression has been found in metaplastic, dysplastic and cancerous gastric tissues but levels were independent of tumour stage, degree of differentiation and tumour type (Ebert *et al*, 2000). In one study, the 5 year survival rate of patients with gastric adenocarcinoma was much poorer in those patients expressing two or more markers of proliferative activity that included MT, glutathione-S-transferase-π or P-glycoprotein (Monden *et al*, 1997). On the other hand, reduced MT expression was found in human colorectal tumours (Duncan and Reddel, 1999), seminomas (Chin *et al*, 1993), hepatocellular tumours (Waalkes *et al*, 1996) and gastrointestinal neoplasms (Janssen *et al*, 2000) and was mainly associated with poor prognosis.

The diagnostic potential of MT in the progression of normal oesophagus through Barrett's to adenocarcinoma has not been elucidated and is the subject of this study.

## METHODS

Samples of approximately 200 mg of normal oesophagus, premalignant tissue (clinically Barrett's oesophagus), carcinoma and normal gastric tissue were taken from 12 patients who underwent oesophagectomy or oesophago-gastrectomy. The distance of the resected tissue from the anatomical gastro-oesophageal junction was determined and the sample was immediately frozen and stored at −70°C until analysed. In a second study, biopsies were taken from 20 patients with a histological record of Barrett's epithelium, and who were undergoing a yearly follow-up to determine the progression of the disease. Nine of these patients were being followed after fundoplication surgery for severe oesophageal reflux. Multiple biopsy specimens were obtained from defined sites of Barrett's columnar epithelium and from macroscopically normal stratified squamous epithelium at least 1–2 cm above the Barrett's epithelium. Biopsy specimens were fixed with 10% neutral buffered formalin and sent for routine histology. A series of fresh biopsy samples was immediately frozen in liquid nitrogen and then stored at −70°C. Tissue and biopsy samples were diluted 1 : 5 with cold homogenate buffer (10 mM Tris-HCl, pH 8.2) and homogenised using a Potter-Elvehjem homogeniser (Wheaton, NJ, USA). The homogenates were then boiled in a water bath for 2 min and then centrifuged for 4 min at 14 000 **g**. Metallothionein was determined by the cadmium-haemoglobin binding assay (Eaton and Toal, 1982). Protein was determined with SERVA-G Coomasie blue dye reagent (Lott *et al*, 1983) using a Cobas Bio Centrifugal Analyser (Hoffman La Roche, Basel, Switzerland) and human serum albumin as standard. Results are reported as the mean±s.e.m. Where appropriate, significance was determined by the Wilcoxon signed-rank test for paired differences, paired *t*-test for two sample means and Student's *t*-test for independent samples. This study was approved by the Research Ethics Committee of the Royal Adelaide Hospital.

## RESULTS

Metallothionein concentrations in the macroscopically normal squamous epithelium were 193±12 pmol Cd bound mg^−1^ protein (*n*=31), 195±28 (*n*=13) and 194±19 (*n*=25) in the upper, middle and lower oesophagus. MT concentration in clinically normal gastric tissue was 446±186 pmol Cd bound mg^−1^ protein (*n*=59). Higher MT levels (*P*<0.001) were found in the upper half of the stomach (489±54, *n*=20) than lower half (148±14, *n*=20) but there was no significant difference between equivalent positions along the greater and lesser curves of the stomach.

In 10 patients with adenocarcinoma (Table 1

**Table 1:**
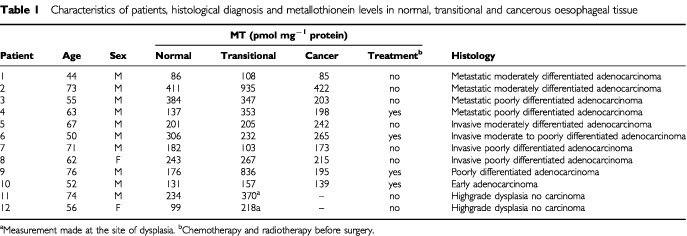
Characteristics of patients, histological diagnosis and metallothionein levels in normal, transitional and cancerous oesophageal tissue

), MT concentrations in metaplastic tissue immediately bordering the adenocarcinoma (354±93 pmol Cd bound mg^−1^ protein) and in adenocarcinoma (214±28) were not significantly different from macroscopically normal oesophagus (226±28). However in the bordering metaplastic tissues of six out of 10 patients with adenocarcinoma and in dysplastic tissue from two other patients, MT concentrations were on average two-fold higher than those in normal oesophagus. The levels of MT in adenocarcinoma were unremarkable and there was no obvious association between the MT content and the histological grading.

Metallothionein concentrations were increased in Barrett's epithelium in 17 out of 20 patients by an average of 108% (Table 2

**Table 2:**
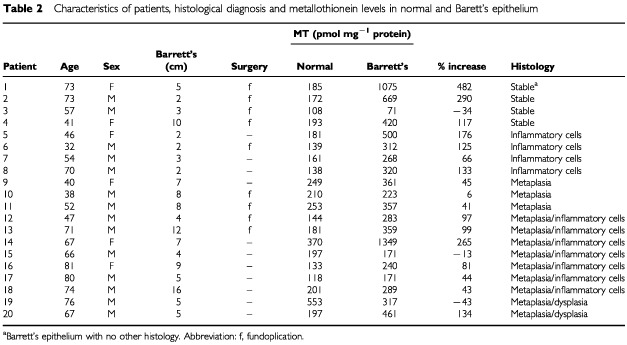
Characteristics of patients, histological diagnosis and metallothionein levels in normal and Barett's epithelium

). The mean MT concentration in normal and Barrett's epithelium was 204±22 (pmol Cd bound mg^−1^ protein; mean±s.d.) and 411±68, respectively. The difference between MT concentrations in normal and Barrett's epithelium was highly significant (*P*<0.004). There was no association between the MT levels in Barrett's epithelium and the histological diagnosis of inflammation, metaplasia or dysplasia.

## DISCUSSION

It has been argued that in Barrett's oesophagus there is a large intra- and interobserver variation in the reporting of various grade dysplasia with inflammatory atypia making the diagnosis problematic. Thus it can be difficult to monitor the progression of oesophagitis to dysplasia in order to detect cancer at a curable stage (Ortiz-Hidalgo *et al*, 1998). This has led to the search for new objective indicators which may complement and help reduce the observer variability with histological diagnosis. MT is a potential marker of carcinogenesis and its expression in human cancers can be up- or down-regulated (Cherian *et al*, 1994). Few studies have focused on the expression of MT in human oesophageal tumours and this is the first to quantitatively measure the concentration of MT in human adenocarcinoma of the oesophagus and in Barrett's epithelium, which is generally considered to be the premalignant lesion, although the primary cells leading to adenocarcinoma have not been identified. In one study, *in situ* hybridisation with MT DNA probes and immunochemistry was used to determine MT mRNA and MT protein expression in resected oesophageal tissue from patients with squamous cell carcinoma. It was concluded that MT expression was a potential marker of the proliferative and metastatic behaviour of this cancer (Hishikawa *et al*, 1997, 1999). Here we demonstrate that MT expression was not increased above matched normal-appearing oesophagus in 10 patients we investigated with adenocarcinoma. In each case, there was histological confirmation that the adenocarcinoma arose from columnar lined Barrett's mucosa. Thus comparison of the MT levels in the adenocarcinoma with those in Barrett's epithelium indicates that MT expression is down regulated in neoplastic progression. Similar changes in MT concentration have been demonstrated in stomach adenocarcinoma. In a study of 35 patients with gastric adenocarcinoma where MT was quantitatively measured on resected tissues, it was found that MT was significantly lower in adenocarcinoma compared to that in normal-appearing gastric mucosa (Janssen *et al*, 2000). In another study on 34 patients with gastric cancer, MT immunoreactivity in the cytoplasm and surface of the tumours was absent or low in 19 cases, moderately increased in 12 patients, and greatly increased in three cases (Ebert *et al*, 2000). There was no association between immunoreactivity tumour stage, grade of differentiation or tumour type. This is not unexpected however, as MT is induced by a variety of inflammatory mediators as well as cytotoxic agents and irradiation. Thus any pre-surgical radiotherapy and/or chemotherapy might affect the level of expression and localisation of MT in these studies. Our finding that MT appears quantitatively higher in the cardia and fundus than in lower regions of the stomach may reflect the glandular composition of these areas. Intense immunoreactivity has been reported in goblet cells and at the foveolar neck of gastric glands (Ebert *et al*, 2000).

Others have demonstrated that MT is responsive to inflammatory and pre-neoplastic changes in normal tissues. In gastric cancer patients, intense immunostaining for MT was found in adjacent metaplastic and dysplastic tissues in 17 out of 34 patients (Ebert *et al*, 2000). Our study of 20 patients with Barrett's epithelium clearly demonstrates that MT is increased in Barrett's tissue above that in normal oesophagus. The underlying reason for this increase remains unknown. There is evidence in mice that overexpression of MT in mutagen-subjected colorectal crypts arises from random stem cell somatic mutation (Jasani *et al*, 1998; Cook *et al*, 2000). Such a mechanism could account for overexpression of MT in Barrett's oesophagus while loss of this gain mutation might occur in those lines of cells that become cancer.

In the present study, there was no obvious association in MT expression between the histological markers of the presence of inflammatory cells, metaplasia or dysplasia. Indeed, two of four patients with histologically stable Barrett's epithelium had the highest levels of MT. This may indicate that MT expression in Barrett's mucosa is more closely related to the higher MT levels found in the specialised mucosa than the pathobiology associated with cancer. Barrett's mucosa is composed of heterogenous cell types. Three histological patterns have been identified including (a) small columnar intestinal-like cells with mucin containing goblet cells and crypt like glands containing Paneth and endocrine cells, (b) fundic mucosa indistinguishable from that in the stomach showing pits with mucous-secreting cells and a few parietal and chief cells and (c) normal gastric cardiac mucosa (Ortiz-Hidalgo *et al*, 1998). Several studies have indicated that the intestinal type with metaplasia is more likely to undergo neoplastic progression than the fundic and cardiac form (Hamilton and Smith, 1987; Filipe and Jankowski, 1993). Interestingly, goblet cells show intense immunostaining for MT (Ebert *et al*, 2000) and may contribute to the higher MT content of the specialised mucosa. In addition, in rats we have shown that MT immunostaining of small intestinal segments is localised to crypt cells and in particular, Paneth cells (Tran *et al*, 1999). Barrett's epithelium is often complex and may contain multiple histological patterns. The finding of high-grade dysplasia in metaplastic epithelium remains the best predictor of increased risk of cancer. Whether MT might prove useful in predicting histological types of columnar change warrants investigation. Immunohistochemical studies will be required to fully identify the specialised cells involved in the increased MT expression. In addition, longitudinal studies on patients with oesophageal reflux without Barrett's epithelium, is required to determine whether MT is a predictor of progression from normal stratified squamous epithelium into Barrett's oesophagus. The level of MT in Barrett's epithelium also might prove relevant to the responsiveness of the oesophagus to pre-surgical radiotherapy and chemotherapy but again this remains to be investigated. For the present we believe the high levels of MT found in columnar metaplastic tissues is an interesting finding which warrants further investigation.
